# Chronische Hepatitis-B-Virusinfektion: aktuelle und zukünftige Therapieansätze

**DOI:** 10.1007/s00103-021-03483-x

**Published:** 2022-01-13

**Authors:** Christoph Neumann-Haefelin, Robert Thimme

**Affiliations:** Klinik für Innere Medizin II, Universitätsklinikum Freiburg, Hugstetter Str. 55, 79106 Freiburg, Deutschland

**Keywords:** Hepatitis-B-Virus (HBV), Direkt antiviral wirksame Agenzien (DAA), Eintrittsinhibitor, Immuntherapie, Therapeutische Vakzinierung, Hepatitis B virus (HBV), Direct-acting antivirals (DAAs), Entry inhibitor, Immunotherapy, Therapeutic vaccination

## Abstract

Zur Therapie der chronischen Hepatitis-B-Virus-(HBV-)Infektion stehen aktuell pegyliertes Interferon-Alpha und Nucleosid‑/Nucleotidanaloga (Entecavir und Tenofovir) zur Verfügung. Diese Medikamente ermöglichen eine Virussuppression und eine Normalisierung des Leberenzyms Glutamat-Pyruvat-Transaminase (GPT) und verhindern ein Fortschreiten der Lebererkrankung. Zahlreiche noch in klinischer Entwicklung befindliche Therapiestrategien haben jedoch eine funktionelle Heilung zum Ziel. Dabei soll erreicht werden, dass das HBV-Hüllprotein HBsAg im Blutserum nicht mehr nachweisbar ist („ausgeheilte“ Hepatitis B). Der vorliegende Beitrag gibt eine Übersicht über aktuelle und mögliche zukünftige antivirale Therapien gegen die chronische HBV-Infektion. Als Grundlage diente eine Literaturrecherche unter besonderer Berücksichtigung der aktuellen Leitlinien sowie aktueller Kongressbeiträge.

Die aktuell verfügbaren antiviralen Therapien führen nur sehr selten zur Elimination von HBsAg (funktionelle Heilung). Auch ist bisher weitgehend unklar, bei welchen Patienten ein Absetzen der Langzeittherapie mit Entecavir bzw. Tenofovir sinnvoll ist. Neue Therapiestrategien in klinischer Entwicklung führen bei einem höheren Anteil der Patienten zur funktionellen Heilung. Wahrscheinlich ist aber eine Kombination mehrerer antiviraler Strategien erforderlich, um die funktionelle Heilung für die Mehrheit der Patienten zu erreichen. Eine solche Therapie kann voraussichtlich in den nächsten 5–10 Jahren vorliegen.

## Abstract (en)

For the treatment of chronic hepatitis B virus (HBV) infection, pegylated interferon-alfa and nucleoside/nucleotide analogs (entecavir and tenofovir) are currently available. These drugs allow viral suppression and normalization of the liver enzyme alanine aminotransferase (ALT) and prevent the liver disease from progressing. However, several therapeutic strategies that are still in clinical development aim at a functional cure. Their aim is that the HBV envelope protein HBsAg is no longer detectable in the serum (“resolved” hepatitis B). This review article provides an overview of current and possible future antiviral therapies against chronic HBV infection. A literature search paying special attention to the current guidelines as well as current conference contributions served as the basis.

The currently available antiviral therapies only very rarely lead to the elimination of HBsAg (functional cure). It is also largely unclear in which patients discontinuation of long-term therapy with entecavir or tenofovir is feasible. New therapeutic strategies in clinical development lead to a functional cure in a higher proportion of patients. However, a combination of several antiviral strategies is likely required to achieve a functional cure in the majority of patients. Such therapies may presumably be available in the next 5–10 years.

## Einleitung

Weltweit sind 300 Mio. Menschen chronisch mit dem Hepatitis-B-Virus (HBV) infiziert und haben ein erhöhtes Risiko für eine fortschreitende Lebererkrankung, die zur Zirrhose sowie einem hepatozellulären Karzinom (HCC) führen kann. So ist HBV zusammen mit dem Hepatitis-C-Virus (HCV) eine der 10 Hauptursachen für die globale Mortalität [1]. Bei rechtzeitiger Diagnose kann mithilfe der aktuell verfügbaren Medikamente HBV jedoch gut kontrolliert und die Entwicklung einer Zirrhose verhindert werden. Zukünftige Therapien zielen auf eine funktionelle Heilung (Functional Cure), die durch den fehlenden Nachweis des HBV-Hüllproteins HBsAg (HBV Surface Antigen) im Blutserum gekennzeichnet ist, mit oder ohne das Auftreten von Antikörpern gegen HBsAg (Serokonversion zu Anti-HBs). Eine funktionelle Heilung macht eine weitere Langzeittherapie überflüssig und führt u. a. zu einem deutlich reduzierten Risiko für ein HCC. Hierfür dürfte jedoch die Kombination mehrerer Therapiestrategien erforderlich sein. In dieser Übersicht werden zunächst die aktuellen und dann die zukünftigen Therapiestrategien gegen die chronische HBV-Infektion dargestellt.

## Aktuelle Therapieansätze

### Therapieziele

Ziele der aktuell verfügbaren Therapieregime sind die Suppression der HBV-Viruslast, die Normalisierung des Leberenzyms Glutamat-Pyruvat-Transaminase (GPT) im Blut, die Regression der Leberschädigung (Fibrosestadium) sowie die Vermeidung von klinischen Endpunkten, also Komplikationen von Zirrhose und HCC [2]. Diese Therapieziele sind bei adäquater Therapieauswahl und entsprechender Compliance bei der großen Mehrzahl der Patienten erreichbar. So ist eine HBV-assoziierte Zirrhose unter konsequenter mehrjähriger antiviraler Therapie sogar reversibel, sofern noch kein Endstadium vorliegt [3, 4]. Bei einem Teil der Patienten mit einem Nachweis von HBeAg (HBV Envelope Antigen), das initial als Marker einer hohen Virusreplikation verwendet wird, kann zudem eine Serokonversion zu Anti-HBe erreicht werden. Eine Elimination des HBsAg, mit oder ohne Serokonversion zu Anti-HBs, wird mit den aktuell verfügbaren antiviralen Medikamenten jedoch selbst bei mehrjähriger Therapie nur bei einem kleinen Teil der Patienten (ca. 10 % nach 5‑jähriger Therapie) erreicht. Diese funktionelle Heilung (Functional Cure) ist jedoch Ziel zukünftiger Therapiestrategien (siehe unten).

### Therapieindikation

Aus dem pragmatischen Therapieziel Suppression der Viruslast und Normalisierung der Transaminasewerte ergibt sich auch die Indikation zur antiviralen Therapie der chronischen HBV-Infektion. Eine eindeutige Therapieindikation ergibt sich dann, wenn die Transaminasewerte erhöht sind und die Viruslast > 2000 IU/ml liegt. Nur mäßig hohe Viruslasten (2000–20.000 IU/ml) erklären allerdings eine Transaminaseerhöhung nicht, sodass nach weiteren Ursachen der Transaminaseerhöhung (z. B. nichtalkoholische Steatohepatitis – NASH, bisher nicht diagnostizierte oder neue Koinfektion mit dem Hepatitis-D-Virus – HDV) gesucht werden sollte.

Eine weitere eindeutige Therapieindikation ergibt sich bei Patienten, bei denen bereits eine Zirrhose (oder fortgeschrittene Fibrose – F3) besteht. Bei diesen Patienten sollte bei positiver HBV-PCR eine antivirale Therapie unabhängig von Transaminasewert und Viruslast eingeleitet werden. Auch Patienten mit HCC sollten, unabhängig von einer kurativen Therapie, bei positiver HBV-PCR antiviral behandelt werden, da dadurch das Rezidivrisiko bzw. der Tumorprogress reduziert werden kann. Eine weitere Therapieindikation sind extrahepatische Manifestationen der HBV-Infektion wie z. B. Polyarteriitis nodosa und membranöse Glomerulonephritis. Die Therapieindikation zur Verhinderung einer HBV-Reaktivierung durch Immunsuppression sowie zur Reduktion des Transmissionsrisikos in der Schwangerschaft werden unten detailliiert beschrieben. Zudem kann aus beruflichen oder sozialen Gründen eine antivirale Therapie zur Reduktion des Transmissionsrisikos indiziert sein.

**Figure 1:**
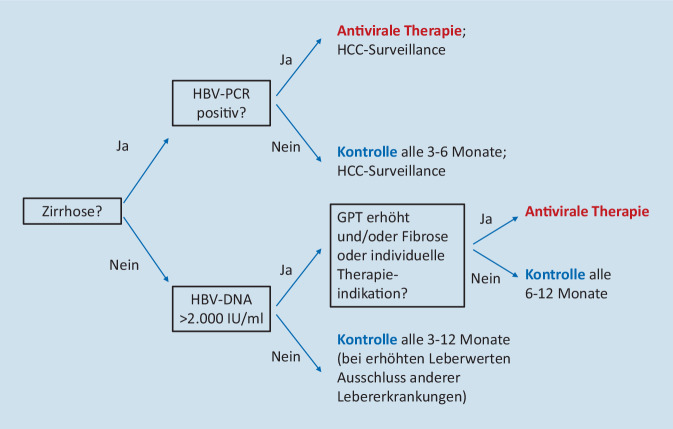

Bei normalen Transaminasewerten und HBV-Viruslast < 2000 IU/ml besteht in der Regel keine Therapieindikation. Diese Konstellation wurde früher als „asymptomatischer HBsAg-Trägerstatus“ bezeichnet (jetzt in Anlehnung an die aktuellen Leitlinien der European Association for the Study of the Liver (EASL) meist als „HBeAg-negative HBV-Infektion“ [5]), da bei diesen Patienten das Risiko einer Progression der Lebererkrankung, das HCC-Risiko sowie die Infektiosität sehr gering sind und von einer antiviralen Therapie keine klinischen Vorteile zu erwarten wären. Kontrovers diskutiert wurde in den letzten Jahren die Therapieindikation bei Patienten mit normalen Transaminasewerten und sehr hoher Viruslast (in der Regel > 1 Mio. IU/ml; früher fälschlicherweise als „immuntolerantes Stadium“ bezeichnet, jetzt „HBeAg-positive HBV-Infektion“ [5]). Hier legt die aktuelle Leitlinie der Deutschen Gesellschaft für Gastroenterologie, Verdauungs- und Stoffwechselkrankheiten (DGVS) eine antivirale Therapie bei einem Alter > 30 Jahren oder hochnormalen Transaminasewerten nahe, da bei höherem Alter und/oder hochnormalen Transaminasewerten das Risiko für Komplikationen wesentlich ansteigt [2]. Abb. 1 zeigt den pragmatischen Algorithmus zur Therapieindikation bei der chronischen HBV-Infektion [2].

### Therapiestrategie

Prinzipiell stehen aktuell 2 Therapiestrategien für die chronische HBV-Infektion zur Verfügung. Pegyliertes Interferon-Alpha 1 × wöchentlich subkutan über 48 Wochen führt bei ca. 20–30 % der Patienten zu einem langfristigen Ansprechen im Sinne einer Reduktion (nicht Negativierung) der Viruslast und Normalisierung der Transaminasewerte. Aufgrund der relativ geringen Ansprechraten sowie potenzieller Nebenwirkungen kommt dieses Therapieregime jedoch aktuell nur bei wenigen Patienten zum Einsatz, v. a. bei jüngeren Patienten mit günstigen Prädiktoren für ein Therapieansprechen (siehe unten).

Die große Mehrheit der Patienten erhält hingegen ein orales Nukleosid- oder Nukleotidanalogon (NUC), welches die HBV-Polymerase inhibiert. Sowohl Nukleosid- (z. B. Entecavir) als auch Nukleotidanaloga (z. B. Tenofovir) konkurrieren mit den natürlichen Substraten der HBV-Polymerase und führen zum Kettenabbruch. Die Therapie mit den modernen NUC Entecavir oder Tenofovir ist gut verträglich und führt zuverlässig zur Suppression der Viruslast und Normalisierung der Transamiansen. Bei Absetzen der NUC kommt es jedoch häufig zum Rebound von Viruslast und Transaminasewerten, sodass die NUC-Therapie langfristig (mehrere Jahre) bzw. bis zur Etablierung neuer Therapieoptionen ggf. sogar dauerhaft gegeben werden muss.

### Diagnostik vor Therapie

Nach Diagnosestellung einer chronischen HBV-Infektion (positives HBsAg ohne Hinweis auf akute Hepatitis, also Transaminasewerten < 5- bis 10facher oberer Normalbereich und anamnestisch kein Hinweis auf kürzliches Infektionsereignis) sollten die HBV-Viruslast und die Transaminasewerte bestimmt sowie eine Lebersonographie durchgeführt werden, ggf. ergänzt durch einen nichtinvasiven Test zur Beurteilung des Fibrosestadiums (z. B. transiente Elastographie), um die Indikation zur antiviralen Therapie zu klären. Zudem sind Koinfektionen mit HCV, HDV und ggf. HIV auszuschließen. Falls die Indikation zur antiviralen Therapie gestellt wird, sollten auch HBeAg sowie Anti-HBe, das quantitative HBsAg sowie, falls eine Therapie mit pegyliertem Interferon-Alpha erwogen wird, auch der HBV-Genotyp bestimmt werden.

### Pegyliertes Interferon-Alpha (PEG-IFN)

PEG-IFN 1 × wöchentlich subkutan über 48 Wochen führt bei 20–30 % der Patienten zu einem andauernden Therapieansprechen im Sinne einer deutlichen Reduktion der Viruslast sowie Normalisierung der Transaminasewerte. Günstige Baselineprädiktoren für ein Therapieansprechen auf PEG-IFN sind deutlich erhöhte Transaminasen, eine niedrige bis mäßig hohe Viruslast (z. B. < 1 Mio. IU/ml) sowie ein HBV-Genotyp A. Während der Therapie kann die Wahrscheinlichkeit eines Ansprechens mittels definierter Stoppkriterien beurteilt werden. Der aussagekräftigste Parameter dabei ist ein Abfall des quantitativen HBsAg zu Therapiewoche 12. Ist zu diesem Zeitpunkt das HBsAg um weniger als 20 % abgefallen oder weiterhin ≥ 20.000 IU/ml (detaillierte Kriterien siehe Leitlinie, [2]), ist ein Therapieansprechen nicht zu erwarten und die Therapie sollte beendet bzw. auf eine orale antivirale Substanz umgestellt werden. Bei der Therapiewahl sind natürlich auch die Nebenwirkungen (u. a. grippeähnliche Beschwerden, psychiatrische Symptome, Leuko- und Thrombopenie) sowie Kontraindikationen von PEG-IFN (u. a. psychiatrische Vorerkrankungen, Zirrhose Stadium Child B/C, Autoimmunerkrankungen) zu beachten.

### Nukleosid‑/Nukleotidanaloga (NUC)

Die beiden NUC Entecavir (ETV; 0,5 mg/Tag bzw. bei NUC-vorbehandelten Patienten 1 mg/Tag) und Tenofovir als Tenofovirdisoproxil (TDF; 245 mg/Tag) sind seit 15 Jahren zur Therapie der chronischen HBV-Infektion zugelassen. Beide NUC sind gut verträglich. Bei der großen Mehrheit der Patienten führen sie zu einer Suppression der HBV-Viruslast in den Bereich der Nachweisgrenze und zu einer Normalisierung der Transaminasen. Die Entwicklung von ETV- oder TDF-Resistenzen kommt in der klinischen Praxis praktisch nicht vor. Ein mangelndes Therapieansprechen ist i. d. R. auf eine mangelnde Compliance bzw. Therapieadhärenz zurückzuführen, was aufgrund der bei HBV-Patienten häufig bestehenden Sprachbarriere ein relevantes Problem darstellen kann.

Wesentliche Langzeitnebenwirkungen sind eine meist langsam progrediente Nierenfunktionsverschlechterung sowie eine langsam progrediente Verminderung der Knochendichte, welche jeweils unter TDF häufiger beobachtet werden als unter ETV. Daher wurde ein neues Tenofovir-Analogon mit größerer Leberspezifität entwickelt: Tenofoviralafenamid (TAF) kann in einer geringeren Dosierung als TDF gegeben werden (25 mg/Tag), erreicht niedrigere Plasmaspiegel bei höheren Gewebekonzentrationen in der Leber und hat daher eine mindestens gleich gute antivirale Wirksamkeit wie TDF bei geringeren Raten einer Nierenfunktionsverschlechterung oder Reduktion der Knochendichte [6, 7]. TAF ist bisher nur als Originalpräparat und nicht als Generikum erhältlich. Eine kosteneffizientere Wahl als der Wechsel von TDF auf TAF bei Nieren- oder Knochendichteproblemen besteht daher im Wechsel auf ETV.

Eine langfristige NUC-Therapie kann nicht nur zur Regression einer Fibrose bzw. Zirrhose führen, sondern reduziert v. a. bei Patienten mit bestehender Zirrhose auch das HCC-Risiko. Dieser Effekt wird etwa 5 Jahre nach Initialisierung der antiviralen Therapie besonders deutlich [8]. Interessanterweise wiesen einige Kohortenstudien darauf hin, dass Tenofovir einen stärkeren Effekt auf das HCC-Risiko hat als Entecavir [9–11], wobei andere Studien diesen Unterschied nicht reproduzieren konnten [12–14]. Somit könnten eventuelle Unterschiede in der Effizienz der beiden Substanzen von der jeweils untersuchten Kohorte abhängen.

### Dauer der NUC-Therapie

Bei initial HBeAg-positiven Patienten kann die NUC-Therapie bei negativer HBV-DNA 12 Monate nach Serokonversion zu Anti-HBe beendet werden. Allerdings tritt eine Serokonversion von HBeAg zu Anti-HBe nur bei einem Teil der Patienten nach mehrjähriger NUC-Therapie ein. Bei initial HBeAg-negativen Patienten ist der einzig definitive Endpunkt einer NUC-Therapie die HBsAg-Negativierung, ggf. mit Serokonversion zu Anti-HBs. In diesem selten erreichten Szenario kann die NUC-Therapie beendet werden. De facto wurde initial HBeAg-negativen Patienten daher eine langfristige Therapie quasi „open end“ oder bis zur Verfügbarkeit neuer Therapieoptionen empfohlen.

Viele Patienten, die bereits seit 5, 10 oder gar 15 Jahren mittels NUC therapiert werden, hinterfragen diese Praxis jedoch zunehmend. Inzwischen liegen die Ergebnisse mehrerer prospektiver, randomisierter und kontrollierter Studien vor, in denen ein Therapiestopp nach langjähriger NUC-Therapie untersucht wurde. Die größte deutsche Studie war dabei die STOP-NUC-Studie [15]. Basierend auf den Studienergebnissen empfiehlt die neue DGVS-Leitlinie, ein Therapieende zu erwägen, wenn die HBV-Viruslast über mindestens 3 Jahre nicht nachweisbar ist, keine fortgeschrittene Fibrose vorliegt und eine engmaschige Nachkontrolle gewährleistet ist [2]. Als zusätzlicher Prädiktor gilt das quantitative HBsAg zum Zeitpunkt des Therapieendes, wobei ein „cut off“ bisher nicht definiert wurde (bei HBsAg < 100 IU/ml hohe Chance eines dauerhaften Ansprechens; bei HBsAg > 1000 IU/ml schlechte Chance).

Die Kriterien zur Indikation einer Retherapie bei Anstieg von Transaminasen bzw. Viruslast entsprechen denen von therapienaiven Patienten. Ein vorübergehender Anstieg der Transaminasewerte könnte dabei jedoch immunologisches Korrelat und Prädiktor einer funktionellen Ausheilung (Verschwinden von HBsAg) sein [16]. Bei einem Anstieg der Transaminasewerte > 5fachem oberen Normalwert sollte jedoch die NUC-Therapie wieder gestartet werden, da bezüglich der Patientensicherheit bei der geringen Evidenzlage kein Kompromiss eingegangen werden sollte. In Einzelfällen wurde nach Beenden der antiviralen Therapie ein abrupter Anstieg der GPT-Werte auf das > 5-Fache der oberen Normgrenze („HBV-Flare“) mit akutem Leberversagen beobachtet, sodass nochmals auf die Notwendigkeit einer guten Patientenaufklärung und engmaschiger Nachkontrollen hingewiesen sei.

### Antivirale Therapie in der Schwangerschaft

Während die Datenlage eine endgültige Beurteilung von ETV noch nicht zulässt, ist TDF (sowie auch die Nukleosidanaloga Lamivudin und Telbivudin, welche zur HBV-Therapie nicht mehr empfohlen werden) in der Schwangerschaft und auch Stillzeit sicher. Eine bestehende ETV-Therapie sollte daher auf TDF umgestellt werden. PEG-IFN ist in der Schwangerschaft kontraindiziert und sollte abgesetzt oder auf TDF umgesetzt werden. Bei hoher Viruslast (> 200.000 IU/ml) sollte eine antivirale Therapie mit TDF möglichst vor der 32. Schwangerschaftswoche, aber nach dem 1. Trimenon gestartet werden, da ansonsten trotz aktiver und passiver Immunisierung bei Geburt ein perinatales Übertragungsrisiko auf das Neugeborene bestehen kann [2]. Durch eine rechtzeitig gestartete TDF-Therapie in Kombination mit der Aktiv-passiv-Immunisierung kann das perinatale Übertragungsrisiko auch bei initial sehr hohen Viruslasten der Mutter auf praktisch null gesenkt werden, wobei der Nutzen bei initial mäßig hoher Viruslast fraglich ist [17, 18].

### HBV-Reaktivierung

**Figure 2:**
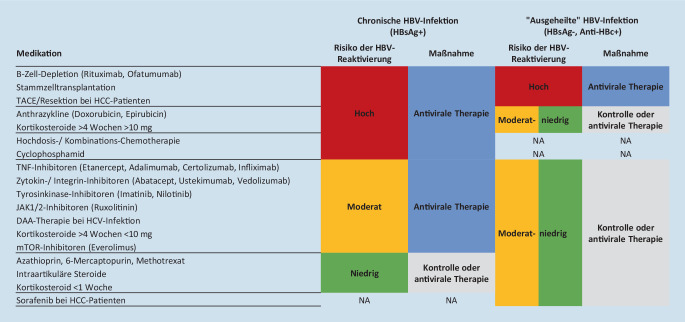

Sowohl bei Patienten mit niedrig-replikativer chronischer HBV-Infektion als auch bei Patienten mit „ausgeheilter“ Hepatitis B (Anti-HBc positiv, HBsAg negativ, Anti-HBs positiv oder negativ) kann es bei starker Immunsuppression zu einer Reaktivierung der HBV-Infektion kommen. Im ungünstigsten Fall steigt die Viruslast dabei auf exzessive Werte an (bis > 40.000.000.000 IU/ml), wobei die Transaminasen typischerweise nur moderat erhöht sind (ca. 100–300 U/L), sich jedoch im Verlauf ein akutes Leberversagen bis hin zum fatalen Ausgang entwickeln kann. Patienten sollten daher vor Start einer entsprechenden immunsuppressiven Therapie auf eine bestehende oder ausgeheilte HBV-Infektion untersucht werden (mindestens Anti-HBc; falls positiv auch HBsAg, Anti-HBs und ggf. HBV-Viruslast). Je nach Risikostratifizierung sollte dann entweder eine antivirale Prophylaxe mit TDF oder ETV oder engmaschige Kontrollen erfolgen. Die neue HBV-Leitlinie der DGVS ist hierbei sehr hilfreich (Abb. 2; [2]). Wichtig ist zu berücksichtigen, dass der immunsuppressive Effekt von Biologicals, wie z. B. Rituximab, oder nach Stammzelltransplantation auch lange nach Therapieende anhalten kann. Nach Rituximab sollte die antivirale Prophylaxe daher über mindestens 18 Monate nach Therapieende fortgeführt werden.

Auch bei erfolgreicher antiviraler Therapie einer HCV-Infektion kann es zu einer Reaktivierung einer zuvor ausgeheilten HBV-Infektion kommen, sodass auch bei diesen Patienten entsprechende Kontrollen während und nach der HCV-Therapie erforderlich sind.

### HCC-Surveillance

Unabhängig von einer antiviralen Therapie haben HBV-Patienten mit einer bereits bestehenden Zirrhose ein deutlich erhöhtes Risiko für ein HCC und sollten daher zur Früherkennung alle 6 Monate mittels Sonographie und ggf. Bestimmung des Alpha-Fetoproteins (AFP) untersucht werden. Doch auch ohne bereits bestehende Zirrhose kann das individuelle HCC-Risiko deutlich erhöht sein. Unter Berücksichtigung der Risikofaktoren ausgeprägte Fibrose (F2/3), Erhöhung der Transaminasewerte, hohe Viruslast (> 2000 IU/ml), positive HCC-Familienanamnese, männliches Geschlecht und Alter ≥ 40 Jahre, HBV-Genotyp C sowie zusätzliche leberschädigende Faktoren (Steatose, Diabetes mellitus Typ 2, Alkoholkonsum, Koinfektionen mit HDV, HCV oder HIV) kann daher auch ohne Vorliegen einer Zirrhose eine HCC-Surveillance empfohlen werden [2]. Eine antivirale Therapie senkt das HCC-Risiko deutlich. Vor allem Patienten mit fortgeschrittener Fibrose sowie Zirrhose haben jedoch selbst nach Elimination von HBsAg weiterhin ein relevantes HCC-Risiko [19].

## Zukünftige Therapien mit dem Ziel der *Functional Cure*

Die aktuell verfügbaren Therapien führen zu einer guten Suppression der HBV-Replikation (gemessen an der HBV-DNA), in der Regel jedoch nicht zu einer relevanten Reduktion der HBsAg-Synthese. Die weiterhin hohen HBsAg-Spiegel verhindern dabei auch eine funktionelle Wiederherstellung der virusspezifischen adaptiven Immunantwort als wichtige Voraussetzungen einer dauerhaften Viruskontrolle. Aktuell sind mehrere neue Therapiestrategien gegen HBV in klinischer Entwicklung, die auf eine funktionelle Heilung, also eine HBsAg-Elimination, zielen. HBV hat sein „Resistenzreservoir“ in Form der sogenannten cccDNA („covalently closed circular DNA“) im Zellkern der Hepatozyten und kann dabei sogar ins Wirtsgenom integrieren [20]. Eine tatsächlich definitive (sterilisierende) Ausheilung mit Elimination der cccDNA sowie der ins Wirtsgenom integrierten Virus-DNA dürfte in absehbarer Zukunft vermutlich nicht möglich werden.

Eine funktionelle Heilung macht eine weitere Langzeittherapie überflüssig und führt u. a. zu einem deutlich reduzierten Risiko für ein HCC. Allerdings bleibt u. a. das Risiko einer HBV-Reaktivierung unter Immunsuppression auch nach funktioneller Heilung bestehen. Daher ist es wichtig zu betonen, dass einer konsequenten HBV-Vakzinierung der Allgemeinbevölkerung (und natürlich insbesondere der Risikogruppen) aus medizinischer und gesundheitsökonomischer Sicht eine besondere Bedeutung zukommt.

Die aktuell in Entwicklung befindlichen antiviralen Strategien lassen sich in 2 Prinzipien unterteilen (Tab. 1; [21]): Die erste Substanzgruppe blockiert spezifische Stufen des viralen „Lebenszyklus“, es handelt sich also um „direkt antiviral wirksame Agenzien“ (DAA). Die zweite Substanzgruppe hat die Aktivierung der antiviralen angeborenen und/oder adaptiven Immunantwort zum Ziel.

**Table 1:** 

Wirkmechanismus	Beispiele für Wirkstoffe (Referenzen siehe Text)
**Direkt antiviral wirksame Agenzien (DAA)**	
Eintrittsinhibitoren	Bulevirtide (BLV)
Kapsid-Assemblierungs-Modulatoren (CAM)	JNJ-6379
HBsAg-Sekretionsinhibitoren	REP-2139, REP-2165
Polymeraseinhibitoren	
Virale Expressionsinhibitoren	
– Small Interfering RNA (siRNA)	JNJ-3989 (ARO-HBV)
– Antisense-Oligonukleotide (ASO)	
**Immunbasierte Therapiestrategien**	
*Aktivierung der angeborenen Immunität*	
TLR8-Agonisten	GS-9688 (Selgantolimod)
*Adaptive Immunität*	
Checkpointinhibitoren	ASC22 (Envafolimab)
Therapeutische Vakzinierung	GS-4774, TG-1050

*TLR8* „toll-like receptor 8“

### Direkt antivirale Agenzien (DAA)

Neue DAA inhibieren entweder den Zelleintritt, die Kapsid-Assemblierung, die HBsAg-Sekretion, die HBV-DNA-Polymerase oder die HBV-Genexpression (Tab. 1).

Der bekannteste *Inhibitor des HBV-Zelleintritts* ist Bulevirtide (BLV; früher Myrcludex B), welches die Bindung von HBV und auch HDV an den Wirtsrezeptor NTCP (Natrium-taurocholat co-transportierendes Polypeptid) und somit die Infektion weiterer Hepatozyten verhindert. Zur Therapie der HBV/HDV-Koinfektion ist BLV bereits zugelassen. In Studien führte es allerdings nur in Kombination mit PEG-IFN bei einem Teil der HBV/HDV-Koinfizierten zu einer Elimination des HBsAg, also einer funktionellen Heilung [22]. In aktuell laufenden Nachfolgestudien bei HBV/HDV-Koinfizierten scheint das Ergebnis bzgl. der Elimination von HBsAg weniger günstig zu sein [23]; Studien bei HBV-Monoinfizierten stehen aktuell noch aus.

Die *Kapsid-Assemblierungs-Modulatoren* (CAM) führen zur fehlerhaften Assemblierung des HBV-Core-Proteins und der HBV-DNA zum Nukleokapsid, wodurch die Produktion infektiöser Viruspartikel, aber auch die Einschleusung von HBV-DNA in den Zellkern und somit die Bildung von cccDNA reduziert wird. Verschiedene Substanzen sind aktuell in klinischer Entwicklung. So führt z. B. JNJ-6379 zu einer signifikanten Suppression der HBV-DNA, nicht jedoch zu einer relevanten Reduktion des HBsAg [24].

Zu den *HBsAg-Sekretionsinhibitoren* zählen die Nukleinsäurepolymere (Nucleic Acid Polymers, NAP) REP-2139 und REP-2165. Diese zeigen zusammen mit PEG-IFN eine beeindruckende Suppression von HBV-DNA und HBsAg sowie eine hohe Rate an funktioneller Heilung (anhaltende HBsAg-Negativität nach Therapieende; [25]). Dabei scheint eine Immunaktivierung mechanistisch beteiligt zu sein, da es bei der Mehrzahl der Patienten zu einem deutlichen Anstieg der Transaminasewerte während der Therapie kommt.

Neue *HBV-DNA-Polymeraseinhibitoren* sollen als Weiterentwicklung der verfügbaren NUC eine noch bessere antivirale Wirksamkeit und Verträglichkeit haben.

Die Substanzen zur *Inhibierung der viralen Genexpression* lassen sich vom detaillierten Wirkmechanismus in kleine eingreifende RNA (Small Interfering RNA, siRNA) sowie Antisense-Oligonukleotide (ASO) unterteilen. Sie führen zu einer Suppression der Genexpression von HBsAg, aber auch von anderen viralen Proteinen. Zahleiche siRNAs und ASO sind aktuell in klinischer Erprobung. So führt z. B. eine Therapie mit der siRNA JNJ-3989 zu einer markanten Reduktion des HBsAg-Spiegels [26].

Gemeinsam ist allen bisher entwickelten DAA, dass sie keine ausreichende antivirale Potenz aufweisen, um alleine zur funktionellen Heilung zu führen. Die Kombination mit PEG-IFN ermöglicht, wie oben für BLV und REP-2139/2165 beschrieben, bei einem substanziellen Anteil der Patienten bereits eine dauerhafte Elimination des HBsAg [22, 25]. Der nächste Schritt auf dem Weg zur funktionellen Heilung als generelles Therapieziel ist jedoch die Kombination von DAA unterschiedlicher Wirkmechanismen. So werden z. B. bei den aktuell laufenden REEF-Studien der Kapsid-Assemblierungs-Modulator JNJ-6379 mit der siRNA JNJ-3989 kombiniert (Studien-Nr. NCT03982186). Die Ergebnisse der Studien werden mit Spannung erwartet. Eine noch effektivere antivirale Wirkung könnte jedoch durch die Kombination der DAA mit der zweiten neuen Therapiestrategie erzielt werden: der Aktivierung der angeborenen und insbesondere der adaptiven Immunantwort [27]. So könnte gerade die Senkung der HBsAg-Spiegel durch DAA eine Aktivierung der adaptiven Immunantwort ermöglichen, die ansonsten aufgrund der hohen Antigenlast erschöpft ist [28].

### Strategien zur Aktivierung des Immunsystems

Eine medikamentöse Aktivierung der angeborenen Immunität ist u. a. durch *Agonisten der Toll-like-Rezeptoren* (TLR) möglich (Tab. 1). Selgantolimod (GS-9688) ist ein TLR8-Agonist und führte in ersten klinischen Studien zu einer guten HBsAg-Reduktion bis hin zur dauerhaften Elimination des HBsAg bei einigen Patienten [29]. Eine weitere klinische Prüfung der Substanz ist u. a. im EU-geförderten Projekt IP-cure‑B geplant (www.ipcureb.eu).

Die adaptive Immunantwort kann zum einen durch *Checkpointinhibitoren* erreicht werden, die sich gegen inhibitorische Immunrezeptoren, wie z. B. PD‑1 oder CTLA‑4, richten und somit die T‑Zellerschöpfung der virusspezifischen T‑Zellen in der chronischen HBV-Infektion reduzieren können. So ist z. B. der PD-L1-Antikörper Envafolimab aktuell in klinischer Erprobung (Studien-Nr. NCT04465890).

Zudem kann die adaptive Immunantwort durch eine *therapeutische Vakzinierung* induziert werden. Bisher wurden in klinischen Studien u. a. Peptidvakzine sowie vektorbasierte Vakzine getestet. GS-4774 ist eine therapeutische Vakzine, die Peptide aus den HBV-Proteinen HBs, Core und X enthält. Eine therapeutische Vakzinierung mit GS-4774 führte zu einer deutlichen Induktion HBV-spezifischer T‑Zellen, allerdings zu keiner signifikanten HBsAg-Reduktion [30]. Eine ähnliche Wirksamkeit zeigte die therapeutische Vakzine TG-1050, die auf einem replikationsdefizienten Adenovirus als Vektor basiert und mehrere HBV-Proteine codiert [31]. Um eine noch bessere Immunogenität zu erreichen und gleichzeitig sowohl virusspezifische Antikörper als auch T‑Zellen zu induzieren, ist in dem EU-geförderten Projekt TherVacB eine therapeutische Vakzinierung mit einem heterologen Prime-Boost-Schema geplant (www.thervacb.eu). Dabei soll die erste Impfdosis mit Proteinvakzine neutralisierende Antikörper und T‑Zellen induzieren, die zweite Impfdosis 4 Wochen später mit einer vektorbasierten Vakzine v. a. die T‑Zell-Antwort weiter boostern. Für eine funktionelle Heilung bei der Mehrzahl der Patienten dürfte jedoch eine Kombinationstherapie z. B. mit DAA und therapeutischer Vakzinierung erforderlich sein.

## Fazit

Die aktuell verfügbaren Therapien der chronischen HBV-Infektion ermöglichen eine Viruskontrolle bei fast allen Patienten, sodass die Entwicklung einer Zirrhose effektiv verhindert werden kann. Auch das HCC-Risiko reduziert sich hierunter deutlich; Patienten mit Zirrhose oder individuellen Risikofaktoren sollten jedoch auch unter Therapie eine regelmäßige HCC-Surveillance erhalten. Bei ausgewählten Patienten kann nach mehrjähriger antiviraler Therapie ein Therapiestopp diskutiert werden, was v. a. bei niedrigem quantitativen HBsAg Erfolg versprechend ist. Aktuell sind zahlreiche neue Therapiestrategien in klinischer Entwicklung, die auf eine funktionelle Heilung, also eine dauerhafte Elimination von HBsAg, zielen. Hierfür wird vermutlich die Kombination unterschiedlicher Therapieansätze erforderlich sein, wie z. B. eine Kombination verschiedener direkt antiviraler Agenzien (DAA) oder die Kombination aus DAA und immunbasierten Therapien, wie z. B. therapeutische Vakzinierung. Aufgrund der hohen Entwicklungsaktivität und erster Erfolge könnten Therapien mit dem Ziel einer funktionellen Heilung durchaus in 5–10 Jahren verfügbar sein.
